# Degradation Performance of Open-Cell Biomaterials from Phosphated Carbonyl Iron Powder with PEG Coating

**DOI:** 10.3390/ma13184134

**Published:** 2020-09-17

**Authors:** Renáta Oriňaková, Radka Gorejová, Martina Petráková, Zuzana Orságová Králová, Andrej Oriňak, Miriam Kupková, Monika Hrubovčáková, Mária Podobová, Matej Baláž, Roger M. Smith

**Affiliations:** 1 Department of Physical Chemistry, Faculty of Science, Pavol Jozef Šafárik University in Košice, Moyzesova 11, 041-54 Košice, Slovakia; radka.gorejova@student.upjs.sk (R.G.); martina.petrakova@student.upjs.sk (M.P.); zuzana.orsagova.kralova@upjs.sk (Z.O.K.); andrej.orinak@upjs.sk (A.O.); 2 Institute of Materials Research, Slovak Academy of Sciences, Watsonova 47, 040-01 Košice, Slovakia; mkupkova@saske.sk (M.K.); mhrubovcakova@saske.sk (M.H.); mpodobova@saske.sk (M.P.); 3 Institute of Geotechnics, Slovak Academy of Sciences, Watsonova 45, 040-01 Košice, Slovakia; balazm@saske.sk; 4 Department of Chemistry, Loughborough University, Loughborough, Leicestershire LE11-3TU, UK; r.m.smith@lboro.ac.uk

**Keywords:** degradable biomaterials, iron, phosphated iron, corrosion behavior, microstructure

## Abstract

Advances in biomedicine and development of modern technologies in the last century have fostered the improvement in human longevity and well-being. This progress simultaneously initiated the need for novel biomaterials. Recently, degradable metallic biomaterials have attracted serious attention in scientific and clinical research owing to their utilization in some specific applications. This work investigates the effect of the polyethylene glycol (PEG) coating of open-cell iron and phosphorus/iron foams on their microstructure and corrosion properties. The addition of phosphorus causes a slight increase in pore size and the deposition of a polymer coating results in a smoothened surface and a moderate decrease in pore diameter. The PEG coating leads to an increase in corrosion rates in both foams and potentially a more desirable product.

## 1. Introduction

The massive evolution of novel technologies enabling the production of advanced biomedical devices with higher clinical performance in the last decades has brought increased living standards and life expectancy [1,2,3]. In addition to permanent medical implants, biodegradable materials have gained substantial interest from the material engineering and medical communities. Temporary intervention is desirable in some specific orthopedic, cardiovascular, and pediatric applications. Biodegradable materials can avoid the negative effects associated with long-term implants, for instance, stress shielding, inflammation, thrombus formation, migration of the implant, and re-intervention to remove devices with a transient function [4,5,6,7,8,9]. Degradable biomaterials represent the next generation of highly bioactive materials, which are envisaged to facilitate the restoration of diseased tissue and thereafter naturally decompose in the human body environment without leaving toxic degradation products, to be replaced by healing tissue [2,6,10,11,12,13]. In orthopedic applications, the degradation rate of an implant should be consistent with the rate of bone tissue reconstruction to maintain the mechanical integrity and to enable a progressive transfer of load to the rebuilding bone. Moreover, the degradation kinetics should be appropriate to prevent an intolerable local or systemic accumulation of degradation products [6,10,11,14,15,16].

Among the three principal bioresorbable metallic elements (Mg, Fe, and Zn), iron and its alloys have received attention as temporary orthopedic implant materials, particularly in appliances necessitating the extensive mechanical support in the course of the bone repairing process [4,6,10,17,18]. Good biosafety and biocompatibility of pure iron have been demonstrated in in vitro and in vivo studies [4,18,19,20,21]. The main drawback of Fe-based materials is that the degradation rate is usually too slow in physiological environments owing to the deposition of the dense film of corrosion products passivizing the surface [4,5,6,10,14,17,18]. Alloying elements, such as Mn, C, Si, and Pd, increase the degradation rate of iron, but at higher concentrations could cause a potentially toxic effect [4,6]. An alternative strategy to improve the kinetics of iron corrosion is modification of the surface and/or structure.

Recently, network-like metal foams [6,17,22,23], including open-cell iron foams [9,11,18], have been identified as promising materials for hard tissue scaffolds as they exhibit a natural bone-like structure, which supports the vascularization, growth, and proliferation of bone cells and reduces corrosion resistance [6,17,18,22,24]. The improvement of the mechanical strength of iron foams can be achieved by the addition of low amounts of phosphorus without retarding the corrosion rate or affecting the cell proliferation [11,14].

The application of biodegradable polymer coatings such as polyethylene glycol (PEG) and poly(lactic-co-glycolic acid) (PLGA) on iron foams represents a new concept to improve the degradation behavior [18]. The local acidity on the iron surface generated by hydrolysis or oxidation of the polymers appears to enhance the degradation process and biocompatibility [18,25,26]. In an earlier paper, we reported that the degradation rate of coated iron foams increased with increasing thicknesses of the PEG layer [25], and in this work, we have set out to extend this study to a thicker PEG coating layer and to include both Fe and Fe-phosphorus (Fe/P) foams. Immersion corrosion tests and potentiodynamic polarization corrosion tests were carried out to study the effect of a thicker PEG layer on degradation rate. Electrochemical impedance spectroscopy (EIS) was selected to identify the interfacial properties of uncoated and coated Fe and Fe/P foams and to investigate in detail the degradation process after the foams were exposed to a simulated body fluid.

## 2. Materials and Methods

### 2.1. Fe Foam Preparation

Carbonyl iron powder (CIP) purchased from BASF (type CC d50 3.8–5.3 μm, Ludwigshafen, Germany) was utilized as raw material. The chemical composition of CIP was: minimum (min.) 99.5% Fe, maximum (max.) 0.18–0.35% O, max. 0.05% C, and max. 0.01% N. Iron foams were manufactured by replication method using polyurethane foam (PUR) (Filtren^®^ TM 25133 with the cell size 1060–1600 µm, Eurofoam, Brno, Czech Republic). The cylindrical specimens (Ø 10 mm, h 20 mm) were cut out from PUR and impregnated with a suspension consisting of 7 g of CIP and 200 mg of gelatin (Sigma-Aldrich, Saint-Louis, MO, USA) in 6 mL of water at 60 °C. The impregnation of PUR foam was conducted for a period of 24 h. Thereafter, the sintering of impregnated cylinders was performed in a tube furnace Aneta 1 (ANETA, Trenčianská Teplá, Slovakia) at 450 °C for 2 h in nitrogen atmosphere to thermally remove the PUR template. The resultant iron foam was produced after subsequent sintering at 1120 °C for 1 h in a reduction atmosphere (90% N_2_, 10% H_2_).

### 2.2. Fe/P Foam Preparation

To obtain the iron-phosphate-coated carbonyl iron powder (Fe/P), a modified precipitation method was applied. The phosphating process was realized by stirring the iron powder in a solution containing acetone and orthophosphoric acid (acetone: H_3_PO_4_ molar ratio of 1:9) for 2 h at room temperature. After that, the phosphated iron powder was dried at 60 °C for 2 h and calcined at 400 °C in air for 3 h. The Fe/P foams were then prepared by the same procedure as Fe foams but to avoid liquid-phase sintering, the Fe/P samples were heat-treated at 1050 °C. The final Fe/P foams contained ~0.5 wt.% of phosphorus. The Fe/P material produced by phosphating comprised of globular iron (*α*-Fe phase) and iron oxide particles, embosomed with a solidified liquid phase consisting of ferric phosphate compounds formed during eutectic reactions in the Fe_2_O_3_–P_2_O_5_ system [27].

### 2.3. Preparation of Polymer Coating Layer

Coating of Fe and Fe/P foams with polyethylene glycol 4000 (PEG) (Sigma-Aldrich, Saint-Louis, MO, USA) was performed through a sol-gel process. Firstly, a 10 wt.% solution of PEG in 96% ethanol (Mikrochem spol. s.r.o., Pezinok, Slovakia) was prepared and held at ambient temperature for 24 h. Then, the foam samples were cleaned by using ultrasound (in acetone, ethanol, and distilled water for 10 min each) and dipped into the PEG solution for 3 h at room temperature, and thereafter, dried for 3 h at 45 °C. To obtain a thicker polymer coating layer compared to earlier work [25], this sol-gel procedure was repeated 3 times.

### 2.4. Material Characterization

The surface appearance and microstructure of the foams was visualized by a scanning electron microscope (SEM) equipped with an energy dispersive spectrometer (EDX) (JEOL JSM-7000F, Tokyo, Japan with EDX INCA).

To determine the specific surface area (*S_BET_*) of the specimens, a low-temperature nitrogen adsorption method using a NOVA 1200e Surface Area and Pore Size Analyzer (Quantachrome Instruments, Hartley Wintney, UK) was applied. The Brunauer-Emmett-Teller (BET) theory was used to calculate the *S_BET_* values.

The FTIR spectra were collected with a Tensor 29 infrared spectrometer (Bruker, Karlsruhe, Germany) using the ATR method in the wavenumber range between 4000 and 650 cm^−1^.

The thermal decomposition of foams was analyzed using a thermogravimetry and differential scanning calorimetry (DSC/TG, JUPITER STA 449-F1 NETZSCH, Selb, Germany) in inert argon atmosphere with a heating rate of 5 °C·min^−1^, up to 1120 °C.

The theoretical sintered density (*ρ_T_*) and total porosity (*π*) of the foams were estimated using gravimetry. To prevent penetration of open pores, the foams were encased in Parafilm and sequentially weighted in air and water at room temperature. The Archimedes’ principle (DIN ISO3369) was used to determine the density from the following equation [28,29]:

(1) $$\rho_{T}=\;\frac{m_{i}}{\frac{m_{PA}-m_{PW}}{\rho_{W}}-\frac{m_{PA}-m_{i}}{\rho_{P}}}$$

where *m_i_*, *m_PA_*, and *m_PW_* are the initial mass (g), mass of the foam encased in Parafilm determined in air (g), and mass of the foam encased in Parafilm determined in water (g), and *ρ_W_* and *ρ_P_* are the density (g cm^−3^) of water and Parafilm.

To calculate the total porosity, the weighting of the foam samples was carried out in air and water after vacuum impregnation with benzyl alcohol. The porosity was determined from the following equation [29]:

(2) $$\pi =\left(1-\frac{m_{i}}{\left(m_{BA}-m_{BW}\right)\;\cdot \;\rho_{T}}\right)\times 100\%$$

where *m_i_*, *m_BA_*, and *m_BW_* are the initial mass (g), mass of the foam impregnated with benzyl alcohol determined in air (g), and mass of the foam impregnated with benzyl alcohol determined in water (g), and *ρ*_T_ is the theoretical density of foam (g·cm^−3^).

### 2.5. Electrochemical Corrosion Test

Electrochemical corrosion studies were performed using an Autolab PGSTAT 302N potentiostat (Metrohm, Herisau, Switzerland). A standard three-electrode electrochemical cell consisted of an iron-based sample as the working electrode, an Ag/AgCl/KCl (3 mol·L^−1^) reference electrode, and a Pt counter electrode. All experiments were carried out with the Hanks’ solution (pH 7.4; 37 ± 2 °C) consisting of 8 g·L^−1^ NaCl, 0.4 g·L^−1^ KCl, 0.14 g·L^−1^ CaCl_2_, 0.06 g·L^−1^ MgSO_4_·7H_2_O, 0.06 g·L^−1^ NaH_2_PO_4_·H_2_O, 0.35 g·L^−1^ NaHCO_3_, 1.00 g·L^−1^ Glucose, 0.60 g·L^−1^ KH_2_PO_4_, and 0.10 g·L^−1^ MgCl_2_·6H_2_O as the simulated body liquid electrolyte.

#### 2.5.1. Potentiodynamic Polarization Tests

The potentiodynamic polarization curves were obtained by varying the applied potential from −400 to −900 mV (vs. Ag/AgCl/KCl (3 mol·L^−1^)) at a scanning rate of 0.1 mV·s^−1^. The corrosion rate (*CR* in mm·year^−1^) was calculated in terms of the corrosion current density (*j_corr_* in µA·cm^−2^) from Equation (3) in accordance with ASTM G59 [30]:

(3) $$CR\;=\;\frac{j_{corr}\;K\;EW}{\rho }$$

where *ρ* is the density (g·cm^−3^), *K* is a constant, which assigns the units for the corrosion rate (3.27 × 10^−3^ for *CR* in mm·year^−1^), and *EW* stands for the equivalent weight (27.92 g·eq^−1^ for Fe). A set of three samples was used in each test.

The corrosion current densities (*j_corr_*), corrosion potentials (*E_corr_*), and corrosion rates (*CR*) were determined by applying the simplified Tafel extrapolation method. For all the potentiodynamic polarization measurements, a delay time of 60 min was set up until the stabilization of the free corrosion potential was achieved.

#### 2.5.2. Electrochemical Impedance Spectroscopy

For the electrochemical impedance spectroscopy (EIS) measurements, the applied frequency range was from 10 mHz to 100 kHz and an alternating current amplitude was ±10 mV. EIS measurements were performed in the potentiostatic mode at a fixed DC potential, which was set at the open circuit potential (OCP).

### 2.6. Immersion Corrosion Test

Before the immersion degradation test, the initial weights of the foams were determined and the samples were cleaned using the ultrasound in acetone and ethanol for 10 min each. Cleaned samples were immersed in 100 mL of the Hanks’ solution for 4, 8, and 12 weeks at 37 ± 1 °C. After the given time of exposure, the samples were removed from the corrosion medium, thoroughly rinsed in water, cleaned using the ultrasound in ethanol for 10 min, and dried. The mass of dried specimens was determined on an analytical balance. The corrosion rate (*CR* in mm/year) was calculated in terms of mass loss from the Formula (4) in accordance with ASTM G31 standard [31]:

(4) $$CR=\frac{8.76\times 10^{4}\;\left(m_{i}-m_{f}\right)}{\rho \;A\;t}$$

where *m_i_* and *m_f_* are the initial mass (g) and final mass after immersion (g), *ρ* represents the density (g·cm^−3^), *t* is the immersion time (hour), and *A* is the exposed surface area of the sample (cm^2^).

## 3. Results and Discussion

Open-cell Fe and Fe/P foams, prepared using previously reported methods [25,27] and coated with a thicker PEG layer compared to our earlier study [25], were examined. The densities of the Fe, Fe-PEG, Fe/P, and Fe/P-PEG foams were 0.72 ± 0.03 g·cm^−3^, 0.95 ± 0.04 g·cm^−3^, 0.81 ± 0.05 g·cm^−3^, and 0.97 ± 0.03 g·cm^−3^, respectively. The amounts of the polymer deposited onto the surface of the Fe and Fe/P foams, calculated from the differences in weight of foams before and after the coating layer deposition, were ~8.5 wt.% and ~8.2 wt.% for the Fe-PEG and Fe/P-PEG samples respectively, higher than the previous samples of 0.8–3 wt.% [25,26,28]. The porosities were 85%, 78%, 80%, and 76%, for Fe, Fe-PEG, Fe/P, and Fe/P-PEG foams, respectively. The values of density, porosity, and macropore diameter are summarized in Table 1.

### 3.1. Surface Morphology and Composition

**Figure 1:**
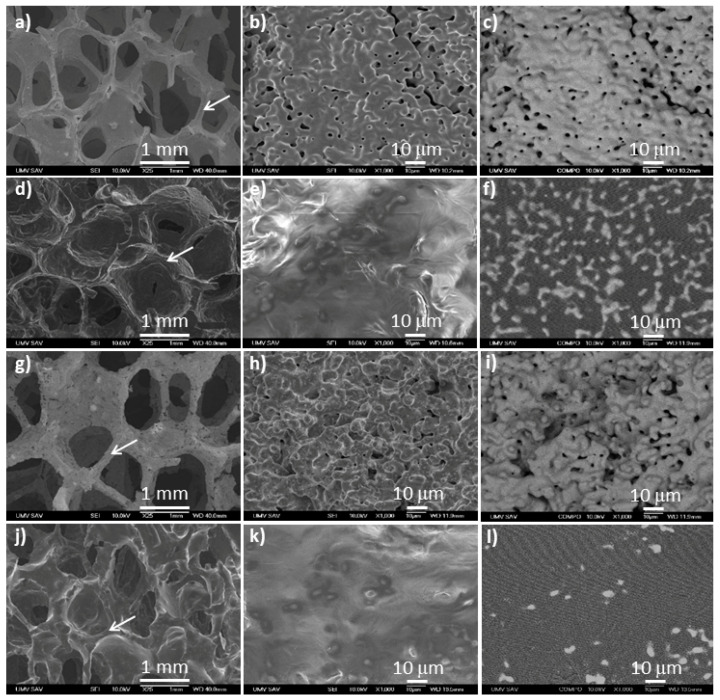
Scanning electron microscope (SEM) micrographs of the surface of iron-based foams: Fe sample (**a**,**b**,**c**), Fe-PEG (**d**,**e**,**f**), Fe/P (**g**,**h**,**i**), and Fe/P-PEG (**j**,**k**,**l**). Magnification: 25× (**a**,**d**,**g**,**j**), 1000× (**b**,**e**,**h**,**k**), and 1000× COMPO mode (**c**,**f**,**i**,**l**).

The SEM micrographs of the surface of iron-based foams are shown in Figure 1 together with details of the surface in secondary electrons (SEI) and backscatter electrons (BSE) (COMPO) imaging regimes.

The arrows in Figure 1a,d,g,j indicate the positions of the higher magnification images. The micrographs showed spherical micropores (up to 5 μm) and macropores (from 400 to 1500 μm) in the structure of sintered foams (25×, Figure 1). While phosphating resulted in a moderate increase in pore size as compared to pure Fe, the PEG coating layer partially filled up the pores on the surface of both Fe and Fe/P samples. The small nodes observed on the surface of Fe and Fe/P samples were covered by the polymeric layer, resulting in a smoothened appearance of the coated samples. This was also reflected in slightly lower values of the specific surface areas (*S_BET_*) of 0.40 ± 0.08 m^2^·g^−1^ and 0.44 ± 0.06 m^2^·g^−1^ for the Fe-PEG and Fe/P-PEG foams compared to 0.48 ± 0.05 m^2^·g^−1^ and 0.50 ± 0.07 m^2^·g^−1^ for Fe and Fe/P samples, respectively.

Higher magnification images of the coated samples revealed that the PEG layer leaked into the pores and flattened the surface roughness (1000×, Figure 1e,k). Also, the COMPO images indicated only isolated islands of Fe (light grey) on the surface of the coated samples protruding from the polymer (dark grey), in contrast to the surface of the uncoated samples which were mainly composed of Fe. Further evidence of the presence of the PEG coating layer was provided by the EDX analysis of the chemical composition of the surface of the sintered samples (Table 2). Carbon and oxygen arising from the PEG layer were detected on the superficial area of the coated foams. The low content of Fe on the surface of these coated samples corresponded to the tips of Fe nodes protruding from the PEG layer.

### 3.2. Thermogravimetric Analysis

**Figure 2:**
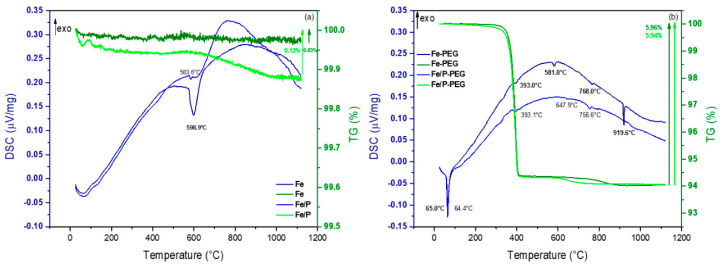
Differential scanning calorimetry/thermogravimetry (DSC/TG) analysis of Fe and Fe/P foams without PEG (**a**) and with a PEG coating layer (**b**).

Another evidence of polymer layer presence on the surface of Fe and Fe/P foams was provided by thermogravimetric analysis. The mass loss and phase transformation according to the DSC/TG measurement of Fe and Fe/P foam without PEG (Figure 2a) as compared to foams with a PEG coating layer (Figure 2b) was evaluated.

The DSC/TG results for Fe and Fe/P foam without PEG showed visible peaks, particularly some ongoing endothermic reactions, not causing a significant mass loss, mainly in the *α*-Fe region (Figure 2a). Behavior of foam samples with a PEG layer during heating is quite different. Even though there is an endothermic peak for Fe foam at 598.9 °C and Fe-PEG foam at 581.0 °C, which are quite close to one another, in the case of the Fe/PEG sample, the mass loss was much higher, approximately 5.9% until the plateau is reached. We can see a similar course of curves accompanied by a significant mass loss in the case of Fe/P and Fe/P-PEG foam samples. This mass loss can be influenced by PEG decomposition. Thermal degradation of PEG started at temperature 65.0 °C for Fe-PEG and 64.4 °C for Fe/P-PEG, corresponding to the endothermic peak, which can represent the melting temperature of PEG 4000 and is related to the mass decrease with the end of this process at about 400 °C, as mentioned in Kou et al. [32]. It can be seen from TG curves that the decomposition of PEG proceeds in a single step, which is in line with results of Pielichowski et al. [33] and Han et al. [34].

The next peak at 581.0 °C for Fe-PEG foam and at 647.9 °C for Fe/P-PEG foam can be related to some chemical reactions between *α*-Fe and products from PEG decomposition. At a temperature of 768 °C (see peak for Fe-PEG foam), iron has a so-called Curie temperature, which means that it no longer exhibits ferromagnetic properties. This is an example of a phase transition. The last significant peak at 919.6 °C (Fe-PEG foam) is a phase transformation from *α*-Fe to *γ*-Fe. In case of the Fe/P-PEG sample, the peaks are shifted towards lower temperatures as compared to the Fe-PEG sample, probably due to the presence of phosphorus from phosphating.

### 3.3. Immersion Corrosion Behavior

**Figure 3:**
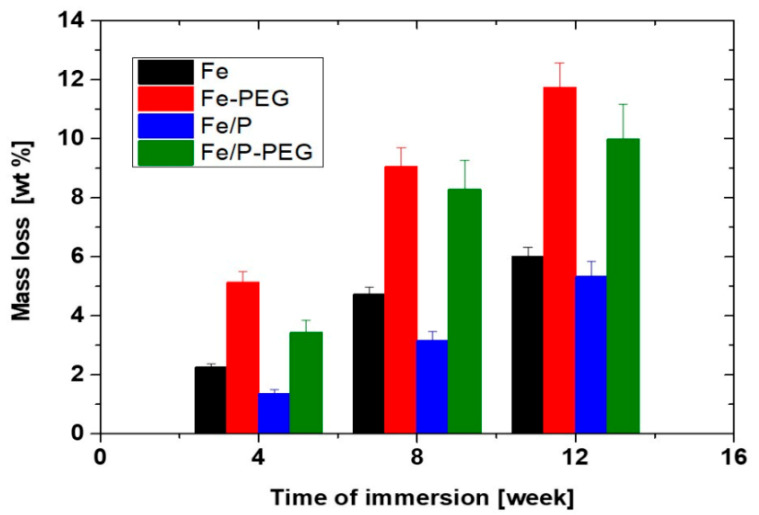
Mass losses during immersion in Hanks’ solution for 4, 8, and 12 weeks for iron-based foams (Fe, Fe-PEG, Fe/P, and Fe/P-PEG).

Mass losses of the studied iron-based foams after immersion in Hanks’ solution during 4, 8, and 12 weeks are displayed in Figure 3. It is notable that mass losses are significantly greater (about 2 times) for foams with a PEG layer as compared to those without a polymer coating. The lowest weight changes were registered for Fe/P foam. However, the difference between mass loss of Fe and Fe/P foam decreased with extension of immersion time. This indicated the higher stability of Fe/P foam in the initial period of corrosion due to the presence of phosphate compounds at the surface of iron particles.

The corrosion rates obtained for iron-based foams in static conditions in Hanks’ solution for 4, 8, and 12 weeks as determined by the mass loss method are presented in Table 3. Phosphating of iron powder caused a slight reduction of corrosion rate of Fe/P foam when compared to the bare Fe foam sample. In consistency with the results of our previous research [25,26,28], the higher corrosion rate was observed for samples with a polymer coating layer as compared to foams without PEG. The higher corrosion rate may be due to both the pH decrease caused by PEG dissolution and degradation and the iron complexation by PEG [28]. The highest corrosion rate was detected for the Fe-PEG sample. Changes in *CR* with time of immersion for samples with polymer were more significant as compared to foams without a PEG coating.

### 3.4. Electrochemical Corrosion Behavior

**Figure 4:**
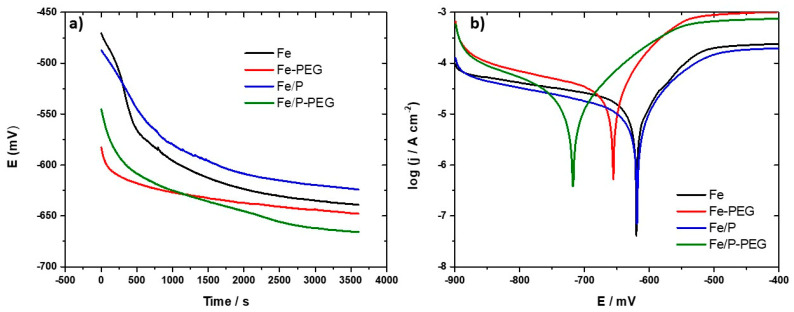
Open circuit potentials (OCP) curves (**a**) and potentiodynamic polarization curves (**b**) of iron-based foams (Fe, Fe-PEG, Fe/P, and Fe/P-PEG) in Hanks’ solution.

To examine the degradation susceptibility of the foams, the open circuit potentials (OCP) time curves were measured in Hanks’ solution (Figure 4a). A decrease of the OCP value was observed for all samples over time. Initial potential values of samples with a PEG coating layer were about 100 mV lower than that of the corresponding samples without a polymeric layer, however the potential drop on the PEG-coated Fe foam was higher than that of the PEG-coated Fe/P sample. This could be related to the appearance of ferric phosphate compounds on the surface of the Fe/P foam sample formed during the phosphating procedure. The phosphating of iron powder and consecutive sintering caused formation of a solidified liquid phase layer consisting of mixed ferric phosphate compounds on the surface of iron powder particles [27,35]. Accordingly, the foam samples manufactured from the phosphated iron powder (Fe/P) exhibited slightly lower corrosion susceptibility as compared with bare iron materials. After an initial period (0–1500 s), the OCP of all foams reached a nearly steady value.

Representative potentiodynamic polarization curves for the foams measured in Hanks’ solution (Figure 4b) were used to determine the corrosion potential (*E_corr_*), corrosion current density (*j_corr_*), and corrosion rate (*CR*) (Table 4).

The corrosion potentials of the pure Fe sample and Fe/P sample were similar, while those of Fe-PEG and Fe/P-PEG samples were shifted toward more negative values. This change implied that the polarization takes place predominantly at the cathode and therefore, that the corrosion of foams with PEG coating layers was cathodically controlled [36]. The corrosion current densities and corrosion rates were in the sequence from high to low: Fe-PEG, Fe/P-PEG, Fe, Fe/P. Acceleration of the corrosion of coated samples can be attributed to local acidity in the vicinity of the surface of coated samples caused by the oxidative degradation of PEG [25,26,28,37]. A decrease in pH increases the solubility of the degradation products and thus hinders their precipitation and enables increased oxygen diffusion [18,25]. Moreover, the local acidic environment enhances the reduction of protons at the cathode, resulting in a higher corrosion current density and therefore in higher corrosion rates [38]. Phosphating the iron powder resulted in moderate retardation of the corrosion rate, which was also observed in our previous work [27]. Coating with PEG led to 80% faster degradation. The degradation of the Fe/P sample was lower but was also increased by 80% in the coated form.

**Figure 5:**
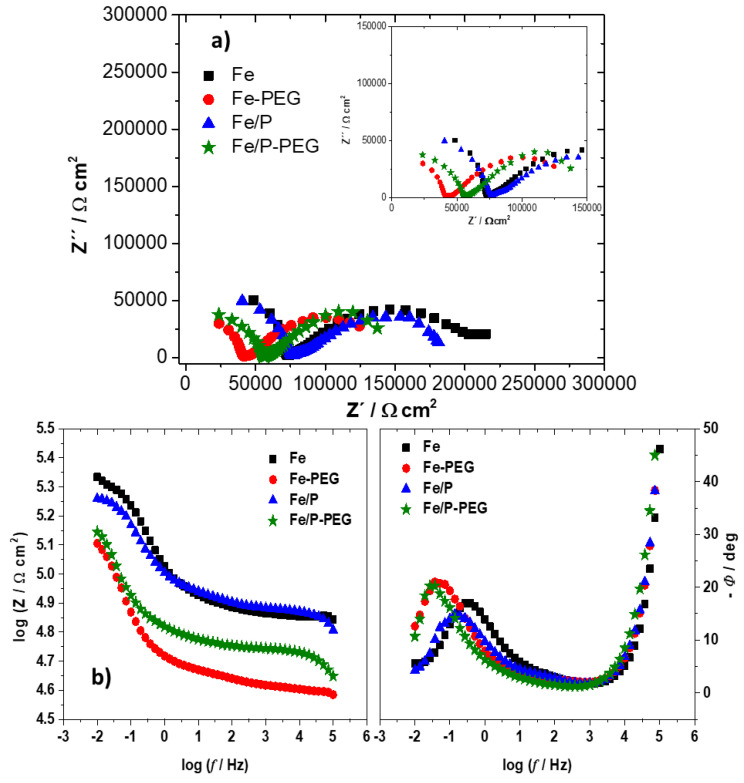
Nyquist diagram (**a**) and Bode diagram (**b**) of iron-based foams (Fe, Fe-PEG, Fe/P, and Fe/P-PEG) in Hanks’ solution.

The results of the electrochemical impedance spectroscopy (EIS) measurements for iron-based foams in Hanks’ solution are shown in Figure 5 as Nyquist plots (a) and Bode plots (b).

Both an incomplete semi-circle in the high-frequency (*f*) region and a flattened loop in the low-frequency region in the Nyquist plots corresponded to capacitive behavior. The depressed appearance of capacitive semi-circle in the low-frequency region reflects the surface inhomogeneity and porosity of sintered foams produced by the powder metallurgy method. The diameters of both semi-circles for Fe and Fe/P foams are larger than for Fe-PEG and Fe/P-PEG foams, which indicates the enhancement of the corrosion susceptibility of the samples with the polymeric layer [39].

The Bode plots exhibit one maximum phase lag at the low-frequency region and a second peak of phase lag arises at the high-frequency region for all foams. The time constant observed in the low-frequency region is related to the charge transfer process of the corrosion reaction. The time constant at the high-frequency region is characterized by the presence of a corrosion product film and different kinds of coating. The intermediate frequency region in the Bode diagram describes the change of electrical conductivity after immersion in corrosive medium [40]. A decrease in impedance value (*Z*) together with the shift of phase shift (*Φ*) to lower frequencies was observed for foams after the addition of P or PEG coating as compared to bare Fe foams (Figure 5b). Values of phase shift were in all cases lower than −45° over almost the whole frequency range, implying the non-uniform distribution of current on the foam surface at this region [41]. The phase shift was higher than −45° only at the highest frequency limit, referring to uniform current distribution. Accordingly, the undeveloped capacitive semi-circle at the high-frequency region could represent the inhomogeneous current spreading on the surface of porous foam samples [41].

The values of charge transfer resistance (*R_ct_*), which corresponds to the polarization resistance, were calculated for all iron-based foams as the subtraction of the impedance values at lower and higher frequencies [6]. The values of double-layer capacitance (*C_dl_*) that correspond to the difference in local charges between the foam sample and electrolyte were calculated using Equation (5):

(5) $$C_{dl}=\sqrt{2\pi \;f_{max}\;R_{ct}}$$

where *f_max_* is the frequency related to the maximal value of the imaginary impedance component (*Z’*). The calculated values of *R_ct_* and *C_dl_* are summarized in Table 5 before and after immersion.

Values of both resistance and capacitance for samples with the PEG coating layer were markedly lower than that for samples without the polymer layer, which is in accordance with results of OCP measurement and potentiodynamic polarization. The higher *R_ct_* values for uncoated Fe and Fe/P foams indicate the formation of a denser passive layer of corrosion products, for example iron oxides, hydroxides, carbonates, and phosphate. The Fe-PEG and Fe/P-PEG foams exhibited lower *C_dl_* values compared to those for Fe and Fe/P foams due to higher dissolution of degradation products in the local acidic environment. This indicated the creation of a less coherent, more porous, and permeable passive layer.

**Figure 6:**
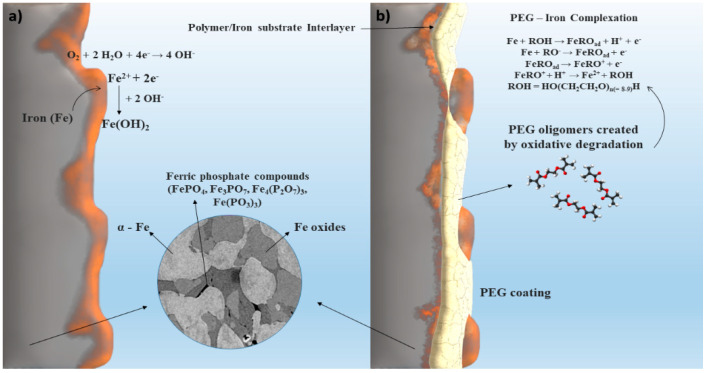
Schematic illustration of differences in passive layer density and corrosion behavior of iron-based foams in Hanks’ solution: Fe, Fe/P (**a**), Fe-PEG, Fe/P-PEG (**b**).

The schematic illustration of differences in passive layer density and corrosion behavior due to the polymer layer is depicted in Figure 6. Degradation of Fe and Fe foam samples proceeded in nearly neutral physiological solution, resulting in formation of a dense layer of corrosion products (Figure 6a). A slight inhibition of corrosion rate at the initial stage due to the phosphating was observed for Fe/P foam. In case of PEG-coated foams, creation of a less dense and leachier passive layer was observed (Figure 6b) owing to the lower pH at the metal–solution interface and iron complexation by PEG. This allowed diffusion of oxygen to the iron surface and rapid degradation of foams.

**Figure 7:**
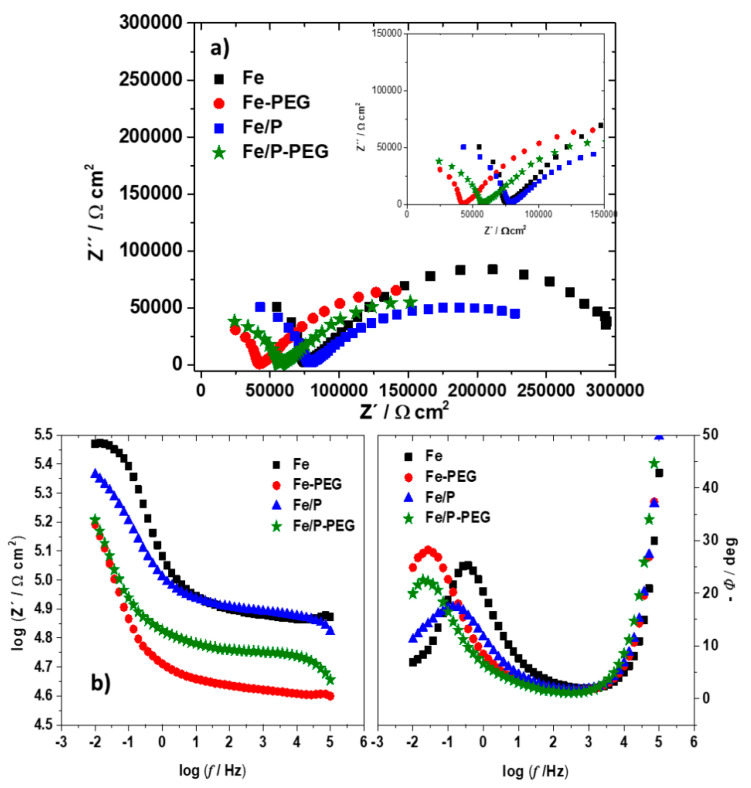
Nyquist diagram (**a**) and Bode diagram (**b**) of iron-based foams (Fe, Fe-PEG, Fe/P, and Fe/P-PEG) after 1 h of immersion in Hanks’ solution.

EIS measurements were also performed after immersion of foams for 1 h in Hanks’ solution (Figure 7). The shapes of the EIS spectra were not changed after exposure in Hanks’ solution (Figure 7), suggesting the same mechanism of corrosion, while the diameter of the depressed capacitive semi-circle in the low-frequency region was higher for all foams, indicating the higher resistance, and thus, the lower corrosion rate [38]. The values of *R_ct_* and *C_dl_* for iron-based foams after immersion were in the same sequence as before immersion. However, the values of *R_ct_* after immersion were approximately 1.2–1.5 times higher than before immersion due to the creation of a degradation products layer. The values of *C_dl_* remained nearly the same before and after exposure to Hanks’ solution, indicating a quasi-steady-state thickness of the adhesive corrosion products layer on the foam sample surface at these conditions.

**Figure 8:**
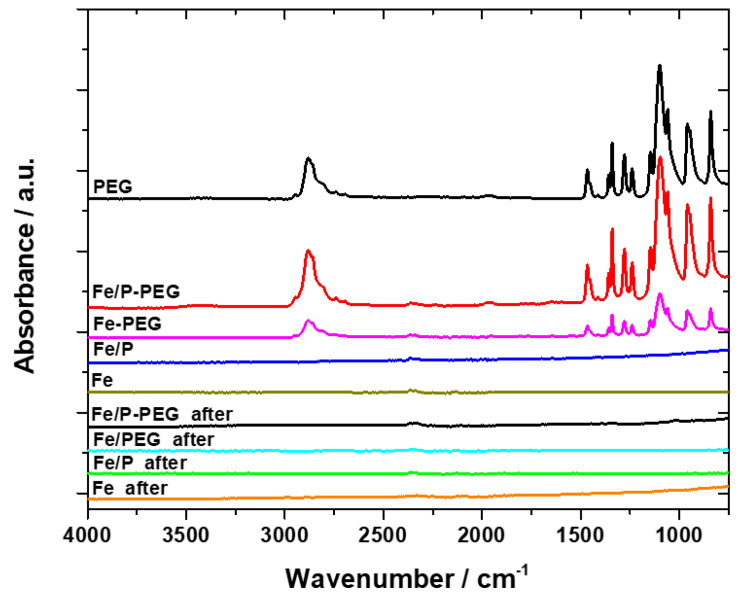
FT-infrared spectra of PEG and iron-based foams (Fe, Fe-PEG, Fe/P, and Fe/P-PEG) before and after 1-week immersion in Hanks’ solution.

The foam samples were investigated by FTIR spectroscopy prior to and after immersion in Hanks’ solution in order to figure out whether or not the PEG polymer was decomposed. The results are summarized in Figure 8.

The reflections of the polymer were found only in the samples before treatment. This is valid for both Fe-PEG samples (with and without P). The peaks of the polymer were at exactly the same positions as in the case of pure polymer, which is in compliance with the results of our earlier research for the coated iron-based foams with the highest PEG concentration [25]. Moreover, the peak at around 876 cm^−1^ was also not observed. The individual vibrations corresponding to various functional groups are described by Shameli et al. [42]. After the 1-week immersion in Hanks’ solution, no signal was detected, pointing to the complete decomposition of the polymer during the immersion. The absence of polymer layer due to its degradation during immersion in physiological solution led to the creation of a denser film of corrosion products associated with higher resistance, and a lower corrosion rate of the foams after the immersion in Hanks’ solution (Table 5, Figure 7).

## 4. Conclusions

Coating of iron-based (Fe and Fe/P) biodegradable materials with a thicker PEG layer than in earlier work was found to produce promising candidates for the design of novel bone substitutes as the procedure increased the degradation rate, which represents the main constraint on the application of iron-based biomaterials. It was thought that local acidity caused by the oxidative degradation of PEG hindered the formation of a compact passive film of corrosion products on the iron surfaces and hence increased biodegradation, which was confirmed by a decrease in polarization resistance detected by potentiodynamic polarization and electrochemical impedance spectroscopy. The values of *R_ct_* and *C_dl_* initially and after 1 h immersion in Hanks’ solution indicated the creation of a more permeable and/or less dense passive layer of degradation products on the surface of coated samples due to the oxidative degradation of PEG. Ions, water, and oxygen could penetrate this layer easier and allow corrosion of the metallic system. The infrared spectroscopy provided proof of the decomposition of PEG during the treatment. The elucidation of the degradation mechanism of these biomaterials in physiological environment demonstrates the possibility of using coating with PEG to control the performance of iron-based degradable biomaterials for healthcare applications.

## Figures and Tables

**Table 1:** Density (*ρ*), total (*π_T_*), open (*π_O_*) and closed porosity (*π_C_*), and pore size (PS) of iron-based foams.

Sample	*ρ* (g·cm^−3^)	*π_T_* (%)	*π_O_* (%)	*π_C_* (%)	PS (μm)
Fe	0.72	85	80	5	400–1500
Fe-PEG	0.95	73	61	12	350–1000
Fe/P	0.81	80	74	6	400–1700
Fe/P-PEG	0.97	70	61	9	350–1100

**Table 2:** Chemical composition of the surface of sintered iron-based foams determined from energy dispersive spectroscopy (EDX) analysis.

Sample	Chemical Composition (wt.%)			
	Fe	C	O	P
Fe	100	0	0	0
Fe-PEG	25.51	46.12	28.37	0
Fe/P	99.53	0	0	0.47
Fe/P-PEG	5.38	53.87	40.75	0

**Table 3:** The values of corrosion rate (*CR*) of iron-based foams (Fe, Fe-PEG, Fe/P, and Fe/P-PEG) immersed in Hanks’ solution during 4, 8, and 12 weeks.

Time of Immersion (Weeks)	*CR* (mm·Year^−1^)			
	Fe	Fe-PEG	Fe/P	Fe/P-PEG
4	0.0229 ± 0.0014	0.0492 ± 0.0020	0.0136 ± 0.0015	0.0415 ± 0.0031
8	0.0231 ± 0.0018	0.0455 ± 0.0036	0.0131 ± 0.0021	0.0397 ± 0.0025
12	0.0222 ± 0.0017	0.0398 ± 0.0028	0.0102 ± 0.0018	0.0364 ± 0.0022

**Table 4:** Electrochemical data and corrosion rate (*CR*) of iron-based foams (Fe, Fe-PEG, Fe/P, and Fe/P-PEG) in Hanks’ solution.

Sample	*E_corr_* (mV)	*j_corr_* (μA·cm^−2^)	*CR* (mm·Year^−1^)
Fe	−620	16.33	0.200
Fe-PEG	−658	29.72	0.362
Fe/P	−617	9.80	0.151
Fe/P-PEG	−718	18.65	0.280

**Table 5:** Impedance parameters of iron-based foams (Fe, Fe-PEG, Fe/P, and Fe/P-PEG) in Hanks’ solution before and after 1-h immersion.

Sample	Before Immersion		After Immersion	
	*R_ct_* (kΩ·cm^2^)	*C_dl_* (μF·cm^−1^)	*R_ct_* (kΩ·cm^2^)	*C_dl_* (μF·cm^−1^)
Fe	146.6 ± 1.7	530.6 ± 6.1	232.1 ± 2.8	671.3 ± 7.8
Fe-PEG	89.28 ± 0.6	155.4 ± 1.3	117.1 ± 1.3	153.5 ± 1.7
Fe/P	105.5 ± 1.5	320.3 ± 4.0	156.8 ± 1.6	396.7 ± 5.0
Fe/P-PEG	87.91 ± 0.9	127.4 ± 1.1	110.3 ± 1.3	129.0 ± 1.3

## References

[1] Li H., Zheng Y., Qin L. (2014). Progress of biodegradable metals, Review. Prog. Nat. Sci. Mater. Int..

[2] Purnama A., Hermawan H., Couet J., Mantovani D. (2015). Assessing the biocompatibility of degradable metallic materials: State-of-the-art and focus on the potential of genetic regulation. Acta Biomater..

[3] Prakasam M., Locs J., Salma-Ancane K., Loca D., Largeteau A., Berzina-Cimdina L. (2017). Biodegradable Materials and Metallic Implants—A Review. J. Funct. Biomater..

[4] Wang S., Xu Y., Zhou J., Li H., Chang J., Huan Z. (2017). In vitro degradation and surface bioactivity of iron-matrix composites containing silicate-based bioceramic. Bioact. Mater..

[5] Heiden M., Walker E., Stanciu L. (2015). Magnesium, Iron and Zinc Alloys, the Trifecta of Bioresorbable Orthopaedic and Vascular Implantation—A Review. J. Biotechnol. Biomater..

[6] Ulum M.F., Arafat A., Noviana D., Yusop A.H., Nasution A.K., Kadir M.A., Hermawan H. (2014). In vitro and in vivo degradation evaluation of novel iron-bioceramic composites for bone implant applications. Mater. Sci. Eng. C.

[7] Hermawan H. (2018). Updates on the research and development of absorbable metals for biomedical applications. Prog. Biomater..

[8] Radha R., Sreekanth D. (2017). Insight of magnesium alloys and composites for orthopedic implant applications—A review. J. Magnes. Alloy..

[9] Sharma P., Pandey P.M. (2019). Corrosion behaviour of the porous iron scaffold in simulated body fluid for biodegradable implant application. Mater. Sci. Eng. C.

[10] Ma J., Zhao N., Zhu D. (2015). Endothelial Cellular Responses to Biodegradable Metal Zinc. ACS Biomater. Sci. Eng..

[11] Yusop A.H., Bakir A.A., Shaharom N.A., Abdul Kadir M.R., Hermawan H. (2012). Porous BiodegradableMetals for Hard Tissue Scaffolds: A Review. Int. J. Biomater..

[12] Čapek J., Vojtěch D., Oborná A. (2015). Microstructural and mechanical properties of biodegradable iron foam prepared by powder metallurgy. Mater. Des..

[13] Sharma P., Pandey P.M.M. (2018). Morphological and mechanical characterization of topologically ordered open cell porous iron foam fabricated using 3D printing and pressureless microwave sintering. Mater. Des..

[14] Wegener B., Sievers B., Utzschneider S., Müller P., Jansson V., Rößler S., Quadbeck P. (2011). Microstructure, cytotoxicity and corrosion of powder-metallurgical iron alloys for biodegradable bone replacement materials. Mater. Sci. Eng. B.

[15] Li Y., Zhou J., Pavanram P., Leeflang M.A., Fockaert L.I., Pouran B., Tümer N., Schröder K.U., Mol J.M.C., Weinans H. (2018). Additively manufactured biodegradable porous magnesium. Acta Biomater..

[16] Ulum M.F., Caesarendra W., Alavi R., Hermawan H. (2019). In-Vivo Corrosion Characterization and Assessment. Coatings.

[17] Hermawan H., Alamdari H., Mantovani D., Dube D. (2008). Iron–manganese: New class of metallic degradable biomaterials prepared by powder metallurgy. Powder Metall..

[18] Yusop A.H.M., Daud N.M., Nur H., Kadir M.R.A., Hermawan H. (2015). Controlling the degradation kinetics of porous iron by poly (lactic-co-glycolic acid) infiltration for use as temporary medical implants. Sci. Rep..

[19] Zhou J., Yang Z., Frank M.A., Detsch R., Boccaccini A.R., Virtanen S. (2016). Accelerated Degradation Behavior and Cytocompatibility of Pure Iron Treated with Sandblasting. ACS Appl. Mater. Interfaces.

[20] Noviana D., Estuningsih S., Paramitha D., Ulum M.F., Hermawan H. (2015). In-vitro Cytotoxicity and In-vivo Tissue Response Study of Foreign Bodies Iron Based Materials. Adv. Mat. Res..

[21] Cheng J., Liu B., Wu Y.H., Zheng Y.F. (2013). Comparative in vitro Study on Pure Metals (Fe, Mn, Mg, Zn and W) as Biodegradable Metals. J. Mater. Sci. Technol..

[22] Stevens M.M. (2008). Biomaterials for bone tissue engineering. Mater. Today.

[23] Lefevre L.-P., Banhart J., Dunand D.C. (2008). Porous Metals and Metallic Foams: Current Status and Recent Developments. Adv. Eng. Mater..

[24] Su Y., Champagne S., Trenggono A., Tolouei R., Mantovani D., Hermawan H. (2018). Development and characterization of silver containing calcium phosphate coatings on pure iron foam intended for bone scaffold applications. Mater. Des..

[25] Haverová L., Oriňaková R., Oriňak A., Gorejová R., Baláž M., Vanýsek P., Kupková M., Hrubovčáková M., Mudroň P., Radoňák J. (2018). An In Vitro Corrosion Study of Open Cell Iron Structures with PEG Coating for Bone Replacement Applications. Metals.

[26] Oriňaková R., Gorejová R., Macko J., Oriňak A., Kupková M., Hrubovčáková M., Ševc J., Smith R.M. (2019). Evaluation of In Vitro Biocompatibility of Open Cell Iron Structures with PEG Coating. Appl. Surf. Sci..

[27] Orinakova R., Orinak A., Giretova M., Medvecky L., Kupkova M., Hrubovcakova M., Maskalova I., Macko J., Kalavsky F. (2016). A study of cytocompatibility and degradation of iron-based biodegradable materials. J. Biomater. Appl..

[28] Oriňaková R., Gorejová R., Orságová Králová Z., Haverová L., Oriňak A., Maskaľová I., Kupková M., Džupon M., Baláž M., Hrubovčáková M. (2020). Evaluation of mechanical properties and hemocompatibility of open cell iron foams with polyethylene glycol coating. Appl. Surf. Sci..

[29] Hrubovčáková M., Kupková M., Džupon M. (2016). Fe and Fe-P Foam for Biodegradable Bone Replacement Material: Morphology, Corrosion Behaviour, and Mechanical Properties. Adv. Mater. Sci. Eng..

[30] ASTM G59 (2009). Standard Test Method for Conducting Potentiodynamic Polarization Resistance Measurements.

[31] ASTM G31 (2012). Standard Guide for Laboratory Immersion Corrosion Testing of Metals.

[32] Kou Y., Wang S., Luo J., Sun K., Zhang J., Tan Z., Shi Q. (2019). Thermal analysis and heat capacity study of polyethylene glycol (PEG) phase change materials for thermal energy storage applications. J. Chem. Thermodyn..

[33] Pielichowski K., Flejtuch K. (2005). Non-oxidative thermal degradation of poly(ethylene oxide): Kinetic and thermoanalytical study. J. Anal. Appl. Pyrolysis.

[34] Han S., Kim C., Kwon D. (1997). Thermal/oxidative degradation and stabilization of polyethylene glycol. Polymer.

[35] Dudrová E., Kabátová M., Hvizdoš P., Oriňaková R. (2015). Sintered composite materials on the basis of Fe/FePO_4_-coated powders. Surf. Interface Anal..

[36] Noor E.A., Al-Moubarak A.H. (2008). Corrosion behavior of mild steel in hydrochloric acid solutions. Int. J. Electrochem. Sci..

[37] Chen B., Evans J.R.G., Holding S. (2004). Decomposition of Poly(ethylene glycol) in Nanocomposites. J. Appl. Polym. Sci..

[38] Wu J., Lu X., Tan L., Zhang B., Yang K. (2013). Effect of hydrion evolution by polylactic-co-glycolic acid coating on degradation rate of pure iron. J. Biomed. Mater. Res. B.

[39] Wang Y.B., Li H.F., Zheng Y.F., Li M. (2012). Corrosion performances in simulated body fluids and cytotoxicity evaluation of Fe-based bulk metallic glasses. Mater. Sci. Eng. C.

[40] Ribeiro D.V., Souza C.A.C., Abrantes J.C.C. (2015). Use of Electrochemical Impedance Spectroscopy (EIS) to monitoring the corrosion of reinforced concrete. Ibracon Struct. Mater. J..

[41] Cheng Q., Chen Z. (2013). The Cause Analysis of the Incomplete Semi-Circle Observed in High Frequency Region of EIS Obtained from TEL-Covered Pure Copper. Int. J. Electrochem. Sci..

[42] Shameli K., Ahmad M.B., Jazayeri S.D., Sedaghat S., Shabanzadeh P., Jahangirian H., Mahdavi M., Abdollahi Y. (2012). Synthesis and Characterization of Polyethylene Glycol Mediated Silver Nanoparticles by the Green Method. Int. J. Mol. Sci..

